# Addressing the Health Needs of People Who Inject Drugs: A Descriptive Analysis of an Inpatient Integrated Care Team Within an Acute Hospital in Scotland

**DOI:** 10.1093/ofid/ofaf147

**Published:** 2025-03-11

**Authors:** Alexandria Chung, Katya Johnson, Bethany Mulloy, Claire L Mackintosh

**Affiliations:** Clinical Infection Research Group, Regional Infectious Diseases Unit, National Health Service Lothian, Edinburgh, UK; University of Edinburgh Medical School, University of Edinburgh, Edinburgh, UK; Emergency Medicine Department, National Health Service Lothian, Edinburgh, UK; Clinical Infection Research Group, Regional Infectious Diseases Unit, National Health Service Lothian, Edinburgh, UK

**Keywords:** assertive outreach, COPAT, integrated care, OPAT, people who inject drugs

## Abstract

**Background:**

People who inject drugs (PWID) experience poor health outcomes secondary to infective sequelae with lengthy hospitalizations, high rates of unplanned discharge, and frequent readmissions. Challenges arranging and engaging with outpatient services compound these issues.

**Methods:**

An integrated team consisting of infectious diseases, drug liaison, and assertive outreach was established in January 2023 at a secondary-care hospital in Edinburgh, Scotland. We assessed the medical needs of patients hospitalized with injection-related infections and considered the impact of this service by comparing health outcomes of patients seen by the integrated team against a historic cohort. Primary descriptive analysis included type and severity of infection, and outcomes included referral to outpatient services and meeting recommended care standards such as blood-borne virus (BBV) screening.

**Results:**

The integrated team saw 37 patients (43 hospital admissions), and 65 patients (73 admissions) were identified as historic controls. Patients seen by the integrated team experienced more severe and complex infections including 37.2% (16/43) of patients having a bacteremia compared to 11% (8/73) in the control group, and a high prevalence of cocaine injection (81% [30/37]). Under the integrated team, higher proportions of patients had a BBV screening (90.7% [39/43] vs 64.4% [47/73]) and were offered outpatient care (81% [35/43] vs 6% [4/73]) with supported attendance.

**Conclusions:**

These results suggest a background of increasingly complicated injection behavior and subsequent infections in Scotland; however, a patient-centered, multidisciplinary care model can effectively address the health need of PWID, offering safer and more appropriate treatment pathways.

Over the past 10 years, hospitalization for the complications of injection drug use (IDU) has been increasing in Scotland [1]. An estimated 1.32% of the Scottish population is directly affected by opioid dependency, amounting to approximately 47 100 people [1]. However, injection behavior has changed in Scotland over recent years including an increased use of cocaine for injection, increased poly-substance use, new synthetic opioids, and more frequent injecting behavior [2, 3]. As well as impacting overdose and mental health–related harms, such injection behaviors also affect hospitalizations secondary to injection-related infections. Such infections may range from deep-seated and hard to manage conditions such as abscesses, infected deep vein thrombosis (DVT), and bacteremia, to blood-borne viruses (BBVs; eg, human immunodeficiency virus [HIV], hepatitis B, hepatitis C) and less complicated skin and soft tissue infections (SSTIs) [4–6]. Further challenges to managing patients with complex drug use often occur in the context of social factors associated with this patient cohort. People who inject drugs (PWID) often experience significant social and medical multimorbidities, which act synergistically to result in higher disease burden rates than the general population [3, 7, 8]. Individuals suffering with addiction and its complications often struggle with prolonged hospitalization, face stigma from healthcare workers, and experience poorly managed symptoms of addiction. This can culminate in high rates of patient-directed discharge and incomplete treatment [9, 10].

In recognition of these multifaceted challenges, the narrative around medical management during hospital admissions for PWID is shifting from disease-focused to person-centered approaches [11, 12]. Recent research has emphasized the benefits of integrated care and considered the impact of interventions such as outreach programs to improve overall patient care and BBV control [13, 14]. When considering the care of PWID admitted with infections, serious injection-related infection (SIRI) teams have been described in the United States (US). These models report higher engagement with opioid replacement therapy and more frequent completion of antibiotic therapy compared to traditional specialty-based care, with good acceptability among patients [11, 15, 16]. However, such teams have so far not been implemented in the United Kingdom (UK), which has a different drug-use landscape and health system compared to the US [17].

Given the growing interest in person-centered approaches to managing injection related infections, our secondary-care hospital established an integrated care team in January 2023. Following a year of its launch, this article aims to evaluate and report on the first cases managed by this integrated team at our hospital in Scotland and explore the role of such a service in the UK healthcare setting.

## METHODS

### Integrated Care Team

The core integrated care team is modeled on the aforementioned US teams and consists of an infectious diseases consultant who advises on infection management; a drug liaison nurse with expertise in substance replacement therapy and harm reduction; an assertive outreach worker whose role is to support patients from hospital stay to community follow-up, including accompanying patients to appointments; and the outpatient antimicrobial therapy (OPAT) service. OPAT and COPAT (complex oral antibiotic therapy) offer ongoing infection specialist management to patients outside the hospital with complex infections requiring antibiotic treatment, either intravenously or oral. Patients are seen daily or weekly for clinical review and drug toxicity monitoring as required. The aims of the integrated care team is to improve the health outcomes of PWID by supporting continued hospital admission where appropriate; supporting safe discharge when a patient-directed discharge occurs; offering infection specialist follow-up and support to attend appointments; improving initiation and retention of medication-assisted treatment for opiate use disorder; and offering harm reduction advice and interventions to bridge the gap between acute hospital and community-led care for this population.

**Figure 1.**
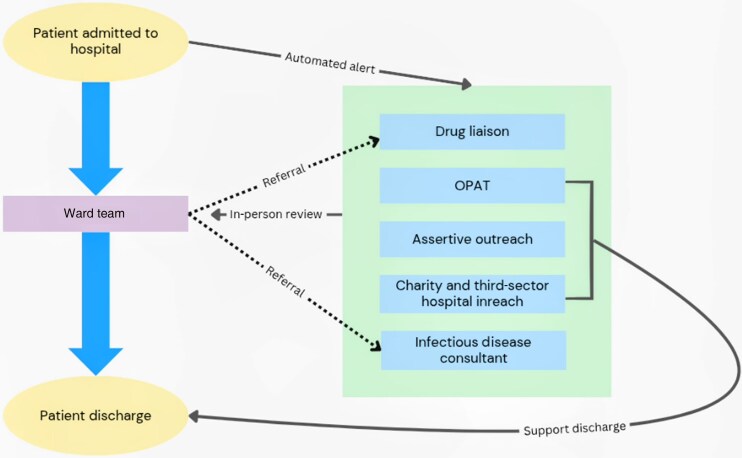
Flow diagram of integrated team communication structure. Image shows the communication structure and interactions of the integrated team in relation to a patient's hospital journey. Abbreviation: OPAT, outpatient antimicrobial therapy.

Figure 1 outlines the communication structure between the core team members, the patient's ward team, and third-sector organizations. On admission, the integrated team receives an automated alert that a potential patient has been admitted to the hospital. Team members will first jointly review the patient and advise on infection management, outpatient treatment plan in the event of a patient-directed discharge, and opiate substitution treatment alongside other harm reduction interventions where appropriate; organize infection outpatient follow-up including transport and logistics; and provide further liaison with third-sector organizations, social workers, housing, and occasionally intermediate care referral. The team corresponds with each other daily via email. In addition, the team meets twice a week in person alongside community outreach pharmacists and specialist homelessness practice nursing staff to discuss all appropriate patients under their care and to smooth barriers between hospital and community care. Alternative routes of communication may be made by direct referrals from the ward team to any member of the integrated team. During a patient's stay in hospital, the assertive outreach worker regularly visits the patient to build a positive rapport and trust, which continues as they transition into community care. The support the assertive outreach worker offers will include accompanying patients to hospital appointments after discharge, liaising with the integrated team on the patient’s behalf after discharge, supporting adherence to oral medications and opiate substitution treatment, and supporting with basic practicalities of living (eg, access to hot food, access to clothing banks, assistance with financial issues, and liaison with third-sector agencies). The duration of outpatient support varies by the needs of the patient and their condition.

A previous audit by our teams had suggested disparities in access to the OPAT service, with those experiencing the highest level of deprivation less likely to be referred than those experiencing the least deprivation [12, 18, 19]. Furthermore, the increased interest in using outpatient services to manage serious infections has been made possible following the pivotal Oral versus Intravenous Antibioitics for Bone and Joint Infection (OVIVA) and the Effficacy and safety of an early oral switch in low-risk *Staphlococcos aureas* blood stream infections (SABATO) studies suggesting that oral antibiotic agents are effective and safe for treating complex infections in selected patients, as well as the introduction of novel long-acting intravenous antimicrobials such as dalbavancin [20–22]. The integrated team follows a harm reduction approach whereby patients are provided treatment without judgment on their drug use behavior and with a focus on removing barriers to access and engagement to care in order to improve clinical outcomes [3, 14]. Beyond the core personnel, the integrated team frequently works alongside other services that are adaptable to the needs of the patient—for example, a BBV team that can evaluate and prescribe relevant hepatitis B and C treatment as well as link the patient with HIV services, social work, and third-sector organizations (eg, homelessness charities).

### Patient Cohorts

Data used in this study were collected as part of routine clinical care. All patients admitted with a drug use–associated infection and reviewed by the newly established integrated team, between 1 January and 31 December 2023, were enrolled into the integrated team cohort. Such patients were initially identified by the drug liaison service through an automated daily generated list that screened all patients admitted to our hospital with key terms related to IDU (PWID [people who inject drugs], IVDU [intravenous drug user]). This list also included patients who were already known to the substance misuse team and individuals with a previous admission related to drug use–associated harms. Patients could also be highlighted to the integrated team via direct referrals from treating clinicians. Patients were excluded from this cohort if they did not have an injection-related infection.

A historic cohort was also identified to understand patient management prior to the implementation of the integrated team. Patients admitted to the accident and emergency department (A&E) between 1 June 2021 and 31 May 2022, identified using the same keywords related to IDU as the intervention cohort, or who were known to the substance misuse team, were screened for an infection-related primary A&E code. Patients were excluded from both cohorts if they met 1 or more of the following criteria: discharged within 24 hours; did not have an infection-related admission; patient denied recent injection, and their primary diagnosis was unlikely to be associated with IDU. Patients in the historic cohort were excluded if they were already identified as part of the integrated team cohort. The period for this historic cohort was chosen to avoid confounding factors associated with the emergency changes in hospital care during the coronavirus disease 2019 pandemic and to prevent misclassification bias while the integrated care team was being set up [23].

### Data Collection

Data were retrospectively collected from the patients’ medical records for both cohorts. Important variables that were collected included patient age and sex, patient self-reported drug use as per A&E notes or drug liaison review, and primary diagnosis. Outcomes of interest included length of admission; whether the patient had taken a patient-directed discharge; whether the patient had been referred for outpatient follow-up relating to their inpatient diagnosis; whether they attended this appointment; and relapse, readmission, or death within 30 days and 90 days of hospital discharge. If a patient was readmitted during the study period, demographic data were taken from only their first admission.

More detailed data from the intervention cohort were collected to further understand this group of patients. Such data included social exclusion, presence of multimorbidity (as defined by having ≥2 chronic conditions), presence of known mental health diagnosis, receiving opiate substitution therapy on admission, review by social worker, and complications associated with their infection. Difficult intravenous (IV) access was defined as the patient either requiring anesthetics input for cannula placement or missing IV antibiotic doses due to not having appropriate IV access at the time of dose administration.

### Ethics and Patient Consent

Data governance was practiced in accordance with the Caldicott principles. Ethical approval was assessed through the National Health Service (NHS) Health Research Authority Decision Tool [24]. As this was a retrospective analysis of routine service data, no formal ethical approval was required, nor was written patient consent required. Access to the final database was restricted to the core team of researchers with specific approvals and only accessible via a secure NHS network.

### Statistical Analysis

Descriptive analysis was used to compare the 2 cohorts. Analysis of data was performed on Stata and R software.

## RESULTS

### Historic Cohort

**Figure 2.**
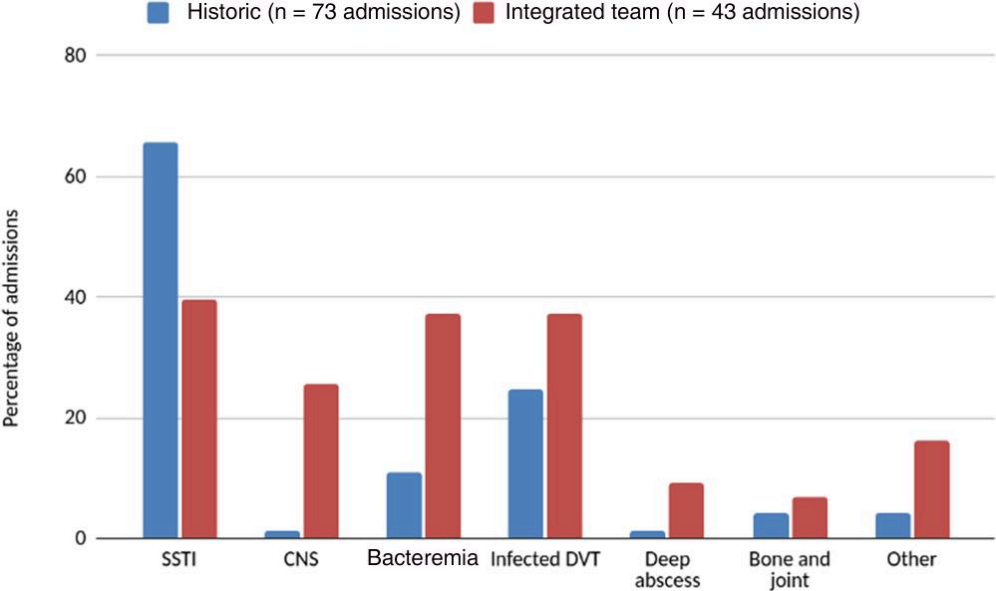
Bar chart showing the percentages of patients within the 2 cohorts who experienced a complication associated with their injection-related infection. Some patients had >1 diagnosis. “Deep abscess” includes retroperitoneal abscess, lung abscess, and psoas abscess. “Other” includes infective endocarditis, ruptured pseudoaneurysm, and dissemination of infected emboli. Abbreviations: CNS, central nervous system infections; DVT, deep vein thrombosis; SSTI, skin and soft tissue infection.

The historic cohort consisted of 65 patients with 73 hospital admissions. The median age of the cohort was 40 years old, with 74% (48/65) of the cohort being male (Table 1). In 2021–2022, heroin was the most commonly used drug. This cohort had a median hospital admission of 4 days (interquartile range [IQR], 2–10 days). During their stay, 66% (48/73) had a BBV screening within the past year. For this cohort, the most common infection presentations were SSTIs (66% [48/73]) (Table 2 and Figure 2).

**Table 1.** Demographic Comparisons of the Historic and Integrated Team Cohorts

Characteristic	Historic (n = 65 Patients)	Integrated Team (n = 37 Patients)
No. of admissions	73	43
Patient characteristics		
Age, y, median (IQR)	40 (35–45)	40 (36–45)
Male sex	48 (74)	26 (70)
Concurrent multimorbidity	NA	6 (16)
Concurrent mental health diagnosis	NA	14 (38)
Drug use		
Heroin	53 (82)	24 (65)
Cocaine	29 (45)	30 (81)
Other drugs	25 (38)	17 (46)
Receiving opioid replacement therapy on admission	NA	22 (59)
Social exclusion		
Homelessness	NA	17 (46)
Other	NA	11 (30)
No. of exclusions per person	NA	0.81

Data are presented as No. (%) unless otherwise indicated.

Abbreviations: IQR, interquartile range; NA, not applicable.

**Table 2.** Comparison of Admission Characteristics by Cohort

Characteristic	Historic (n = 73 Admissions)	Integrated Team (n = 43 Admissions)
Admission data		
BBV screening^a^	48 (66)	39 (91)
Infection specialist review	16 (22)	43 (100)
Length of admission, d, median (IQR)	4 (2–10)	18 (7–31)
Contact with drug liaison	NA	42 (98)
Contact with social worker	NA	17 (40)
Contact with infection service	NA	43 (100)
Contact with assertive outreach	NA	20 (47)
Discharge data		
Patient-directed discharge	22 (30)	10 (23)
Readmission at 30 d	23 (32)	11 (26)
Readmission at 90 d	23 (32)	22 (51)
Death within 3 mo	0	0
Outpatient follow-up offered	4 (5)	35 (81)
Outpatient follow-up attended	3 (4)	24 (56)

Data are presented as No. (%) unless otherwise indicated.

Abbreviations: BBV, blood-borne virus; IQR, interquartile range; NA, not applicable.

^a^This refers to having had a human immunodeficiency virus serology test or hepatitis B and hepatitis C antigen test within 12 months of admission.

Regarding posthospital care, the historic cohort had virtually no outpatient follow-up. Readmission at 30 and 90 days occurred in 32% (23/73) of patients. Of this cohort, 30% (22/73) had a patient-directed discharge; however, no patients died during the observation period.

### Integrated Team Cohort

In 2023, 37 patients were seen by the integrated team over 43 hospital admissions. Similar to the historic cohort, the group of patients seen by the integrated team had a median age of 40 years and 70% (26/37) were male (Table 1). More detailed demographic analysis of this cohort suggested that a high proportion of the patient group experienced a detrimental social determinant of health, with, notably, 46% (17/37) experiencing homelessness. This cohort also reported a high proportion of mental health diagnoses (38% [14/37]) and multimorbidity (16% [6/37]). Interestingly, 81% (30/37) of the intervention population reported using cocaine while 65% (24/37) reported heroin use (Table 1). A high proportion of patients also reported poly-substance misuse including benzodiazepine and gabapentin; however, we did not have further information on how these substances were being consumed. On their first admission, 59% (22/37) of the cohort reported receiving regular opioid replacement therapy; however, by the end of their admission, 83.8% (31/37) were prescribed some form of regular opioid replacement therapy. Reasons for not being on replacement therapy included the patient not wanting to be prescribed replacement therapy or replacement therapy being initiated after discharge.

As with the historic cohort, a significant proportion of patients experienced SSTI; however, there appeared to be a higher proportion of patients in this group who experienced deeper and more complicated infections such as an epidural abscess, infected DVT, and bacteremia (Figure 2). A more detailed analysis of these complications shows that 35% (13/43 admissions) required surgical intervention and, notably, 65% (28/43) of patients encountered difficulties with their IV access, which resulted in delayed or missed doses, and may have required assistance from the anesthetics team. The integrated team cohort had no cases of HIV or hepatitis B infection detected during BBV screening; however, 11 cases of active hepatitis C were identified, some of whom were already undergoing treatment and some of which were new diagnoses. In comparison, of the 48 patients who were screened for a BBV in the historic cohort, 3 tested positive for active hepatitis C.

The median length of admission for patients seen by the integrated team was 18 days (IQR, 7-31 days), suggesting very variable lengths of stay. Regarding readmission, 26% of episodes resulted in readmission at 30 days and 51% were readmitted at 90 days. There were 10 patient-directed discharges, and no patients died during the observation period. Outpatient follow-up was offered on 35 occasions, of which 24 individuals attended (Table 2).

## DISCUSSION

Our study highlights the significant health burden faced by people admitted with injection-related infections, how this burden has changed in a short space of time, and how the implementation of an integrated care team can reduce the likelihood of an unplanned discharge, improve engagement with outpatient care, and potentially offer safe alternatives to admission in the event of a patient-directed discharge.

The age and sex distribution of the 2 cohorts was similar (Table 1). However, the nature of infections experienced by the 2 groups differed, with the historic cohort experiencing fewer complicated infections than the cohort seen by the integrated team. Given the small number of patients, however, statistical significance could not be determined. On the other hand, the primary infection profile does not account for the differences in BBV screening, with 66% (48/73 episodes) of the historic cohort having undergone BBV screening within 12 months of admission compared to 90.7% (39/43 episodes) of those seen by the integrated team (Table 2). Notably, the intervention group was more frequently offered outpatient follow-up than their historical counterparts.

### Patient Cohort and Infection Risk

Although our study lacked objective laboratory evidence to compare injection behaviors between 2021–2022 and 2023, the self-reported drug use suggests that some individuals may have transitioned from using heroin as their main drug of choice to using cocaine (Table 1). These findings correspond with trends seen in other parts of Scotland—namely, both an increase in poly-substance injection and an increased use of cocaine as the solely injected drug [2, 17]. This pattern of drug use differs from that encountered by the SIRI teams in the US who most commonly manage heroin addiction [16, 17]. The shift from predominantly using opioids to cocaine is significant as there are fewer replacement therapies and overdose treatments for the latter drug. Moreover, people who inject cocaine tend to do so more frequently than heroin, which increases the likelihood of vascular and soft tissue trauma and ultimately infection [2, 17, 25]. Last, given the known increased cardiovascular and stroke risk posed by cocaine use, this trend could result in theoretically higher rates of neurovascular events and subsequent significant postevent morbidity in this population [26]. Unfortunately, there are few data in the literature on the correlation between cocaine use and the risk of developing different types of infections; however, the association between its vasoconstrictive and tissue-damaging effects and central nervous system (CNS) infections could be an area warranting further investigation.

### Person-Centered Care

Our analysis suggests that patients seen in 2023 were being admitted for around 14 days more than their counterparts in 2021–2022. These patients experienced high rates of complications and more complex infections such as bacteremia and CNS infections. The relationship between the complexity of infections and admission length is direct: Complicated infections require longer courses of treatment. A less obvious reason for the increase in admission length may be due to the more person-centered approach offered by the integrated health team, resulting in patients being more engaged with health services [11]. Reducing access barriers and encouraging person-centered care alongside building trust with outreach workers may account for the higher rates of referrals to OPAT and the higher subsequent attendances [3, 11, 12]. Notably, enhanced postdischarge care may also partly explain the higher proportion of day 90 readmissions in the intervention cohort where patients were more closely monitored by medical staff and therefore more quickly referred to acute services in the event of a clinical deterioration or concern.

Given the heterogeneity in personal circumstances, infection complications, and drug-use behaviors, there has been much focus in recent years on person-centered and integrated care models for the PWID population [16, 27–29]. The key components of our integrated care team, which differ from previous care models in Scotland, have been the additional support of an assertive outreach worker providing support to the patient across the secondary-care community interface, the extension of our OPAT service to actively include more vulnerable patients, and a formalized communication structure between the multidisciplinary team. Informal feedback from our patient cohorts suggests that the keystone to service acceptability and engagement is the role of our assertive outreach worker in providing continuity of care between the hospital and the community and being a “familiar face” they can trust [3].

Last, evidence suggests that there is a gap between the needed amount of appropriate posthospitalization outpatient care and that which is offered, especially when inpatient care plans are interrupted through patient-directed discharges. Work describing poor health outcomes among patients who repeatedly miss hospital outpatient and primary care appointments shows a correlation between an irregular discharge from hospital and poor attendance at outpatient appointments [30]. This work, and previous studies, also suggest that in addition to vulnerable patients struggling to attend outpatient care, these services are also not being offered to this population [7, 12]. This gap between need and provision is often filled by an increase in unplanned hospitalizations and an increase in all-cause mortality. This bears high health and economic costs to both patients and the health system [30, 31]. As such, individualized and patient-centered interventions that increase referral and attendance at appropriate follow-up and outpatient services should be explored further to mitigate the use of unplanned and emergency services [3, 7, 29]. By supporting patients through a hospital stay and providing as safe as possible discharge with support from an outreach worker, we were able to demonstrate high retention in outpatient care and continued specialist infection input while maintaining patient choice and autonomy [10].

### Limitations and Further Work

Although the model on which we based our integrated team is relatively new and therefore the required specialties to define such a team vary, we recognize that the breadth of our multidisciplinary team may be hard to replicate in smaller or less well-resourced settings [15]. However, the crux of our team revolved around increasing cross-disciplinary and relational communication, by bringing together several preexisting services into a more formal and coordinated structure. Arguably, the definition of an integrated care team may not so much depend on a prescribed list of represented parties, but the processes by which these parties work together and communicate.

When considering our study limitations, longer follow-up periods and larger cohorts would have helped us derive enough statistical power to infer more substantial conclusions. However, as the team's working practice becomes more streamlined and an awareness of its existence grows among acute hospital specialties and patients, we hope to report on other important factors and long-term outcomes such as 6- to 12-month acute hospital readmission rates and clinical outcomes. Specifically, understanding the changes in injecting behavior and what additional factors may be contributing to changes in infection presentation will be crucial in allowing us to tailor patient-centered interventions [3, 32]. Moreover, to understand drivers for patient-directed discharges and frequent readmissions, further research can focus specifically on this group's needs and profile in the UK setting [2, 3, 11], especially with further pilot programs of integrated models in different settings [28].

## CONCLUSIONS

This study has highlighted the high infection-related health needs of a vulnerable and multimorbid population that is not captured in routine national health data. The study suggests that the implementation of an integrated infection-drug liaison team with embedded assertive outreach is feasible and improves engagement with care and specialist infection follow-up. However, the impact on factors such as 30- and 90-day readmission rates remain inconclusive. Accordingly, this model may help to facilitate the meeting of recommended care standards such as increased BBV screening, increasing the use of outpatient and community-led care, and providing improved continuity of care [3].

Unfortunately, the population suffering with addiction-related morbidity faces several challenges in the imminent future, such as the increased prevalence of synthetic drugs (eg, nitizine) across the UK and Europe [17], as well as worsening levels of poverty and health inequality exacerbating the drivers of ill health [33, 34]. These are further compounded by continued financial pressures on health systems, which threaten to impact service capacities [34]. Considering this sociological and epidemiological backdrop, we may expect to see further and more complicated drug-related admission to hospital. Thus the need for novel strategies to better care for patients, such as the creation of integrated care teams, is urgently and pertinently required [35].

## Notes

***Financial support.*** Funding for open access fees has been granted by the Clinical Infection Research Group, National Health Service Lothian, Edinburgh, UK.
